# Sodium Salicylate Influences the *Pseudomonas aeruginosa* Biofilm Structure and Susceptibility Towards Silver

**DOI:** 10.3390/ijms22031060

**Published:** 2021-01-21

**Authors:** Erik Gerner, Sofia Almqvist, Peter Thomsen, Maria Werthén, Margarita Trobos

**Affiliations:** 1 Department of Biomaterials, The Sahlgrenska Academy, University of Gothenburg, 405 30 Gothenburg, Sweden; peter.thomsen@biomaterials.gu.se (P.T.); werthenm@gmail.com (M.W.); 2 Mölnlycke Health Care AB, 415 02 Gothenburg, Sweden; sofia.almqvist@molnlycke.com; 3 Center for Antibiotic Resistance Research (CARe), University of Gothenburg, 405 30 Gothenburg, Sweden

**Keywords:** *Pseudomonas aeruginosa*, quorum sensing, wound infection, biofilm, sodium salicylate, silver

## Abstract

Hard-to-heal wounds are typically infected with biofilm-producing microorganisms, such as *Pseudomonas aeruginosa,* which strongly contribute to delayed healing. Due to the global challenge of antimicrobial resistance, alternative treatment strategies are needed. Here, we investigated whether inhibition of quorum sensing (QS) by sodium salicylate in different *P. aeruginosa* strains (QS-competent, QS-mutant, and chronic wound strains) influences biofilm formation and tolerance to silver. Biofilm formation was evaluated in simulated serum-containing wound fluid in the presence or absence of sodium salicylate (NaSa). Biofilms were established using a 3D collagen-based biofilm model, collagen coated glass, and the Calgary biofilm device. Furthermore, the susceptibility of 48-h-old biofilms formed by laboratory and clinical strains in the presence or absence of NaSa towards silver was evaluated by assessing cell viability. Biofilms formed in the presence of NaSa were more susceptible to silver and contained reduced levels of virulence factors associated with biofilm development than those formed in the absence of NaSa. Biofilm aggregates formed by the wild-type but not the QS mutant strain, were smaller and less heterogenous in size when grown in cultures with NaSa compared to control. These data suggest that NaSa, via a reduction of cell aggregation in biofilms, allows the antiseptic to become more readily available to cells.

## 1. Introduction

Approximately 1.5% of the population in the Western world will suffer from hard-to-heal wounds [1], and the presence of biofilms in these wounds is among the most important contributors to delayed healing [2]. Aggregated bacteria in biofilms are substantially more tolerant to antimicrobial agents and the host immune system than their planktonic counterparts due to protective biofilm matrix components, such as proteins, polysaccharides, and extracellular DNA (eDNA) [3]. A recent systematic review and meta-analysis revealed that a majority of chronic wounds (78%) contain biofilms [4], which are believed to contribute to delayed wound healing and should, therefore, be targeted when treating chronic wounds [2]. Alongside debridement and cleaning of the wound, the topical or systemic administration of antimicrobials can be used to combat biofilm-associated infections [2]. However, due to the high antimicrobial tolerance of cells in biofilms [5] and the risk of antimicrobial resistance development, which is estimated to cause more yearly deaths than cancer by 2050 [6], alternative treatment strategies are needed.

The common wound pathogen *Pseudomonas aeruginosa*, which is frequently isolated from chronic wounds [7], typically produces biofilms and a wide range of tissue-damaging virulence factors and is associated with large and growing wounds [8]. Quorum sensing (QS), a bacterial communication system that regulates important virulence factors [9,10] and biofilm formation [11,12], has long been an attractive target in the search for new antimicrobial treatment strategies. In *P. aeruginosa*, three different but interconnected QS systems (*las*, *rhl*, and *pqs*) regulate, together with their respective QS signal molecules, the production of a wide array of virulence factors [9]. The *las* system regulates virulence factors, such as elastase and alkaline protease, while *rhl* controls rhamnolipid and pyocyanin production and is also affected by the *pqs* system. Both *rhl* and *pqs* are positively regulated by the *las* system. While *pqs* induce the *rhl* system, it is itself reduced by the same, making *pqs* an important regulatory link between *las* and *rhl* [9]. The first study reporting QS-dependent changes in biofilm development showed that a strain defective in the *las* system produced flat homogenous biofilms which were more easily eradicated with detergents than its parent wild-type strain [11]. Since then, QS has been shown to promote biofilm formation via the production of several biofilm components, such as rhamnolipids, lectins, eDNA, and polysaccharides [13]. Numerous efforts have been made to identify effective quorum sensing inhibitors (QSIs) for clinical use as an alternative to traditional infection control strategies. Although many substances have been shown to attenuate QS, biofilm development, and the production of virulence factors in vitro and in vivo, very few have been evaluated clinically [14], and, to the best of our knowledge, none has yet reached clinical practice. QSIs could potentially be used as either a stand-alone treatment, relying on the immune system for infection clearance, or in combination therapy with antiseptics or antibiotics for synergistic effects [15].

Acetylsalicylic acid (Aspirin™) and its active metabolite salicylic acid have both been shown to attenuate QS in *P. aeruginosa*, resulting in reduced virulence factor production and biofilm formation [16,17,18]. However, from a clinical perspective, there is a need to evaluate QSIs in more realistic wound-like environments. Our group previously showed that the sodium salt of salicylic acid (sodium salicylate, NaSa), which is 100-fold more soluble than acetylsalicylic acid [19], modulates QS in *P. aeruginosa* laboratory strains and chronic wound isolates, resulting in the decreased production of several virulence factors [20]. Considering its similarities to acetyl- and salicylic acid, NaSa is an interesting candidate to further assess for its potential anti-biofilm activity. In the present study, silver was chosen as a model antimicrobial agent for use in combination with NaSa because of its regular use in wound care, with silver salts often being incorporated into wound dressings [21].

The aims of the present study were (i) to investigate the effects of NaSa on the biofilm formation and architecture of *P. aeruginosa* and (ii) to evaluate the combined antimicrobial effect of sodium salicylate and silver in a serum-containing wound-relevant 3D biofilm model [22].

## 2. Results

### 2.1. Increased Silver Susceptibility of Biofilms Formed by Pseudomonas aeruginosa in the Presence of NaSa

**Figure 1:**
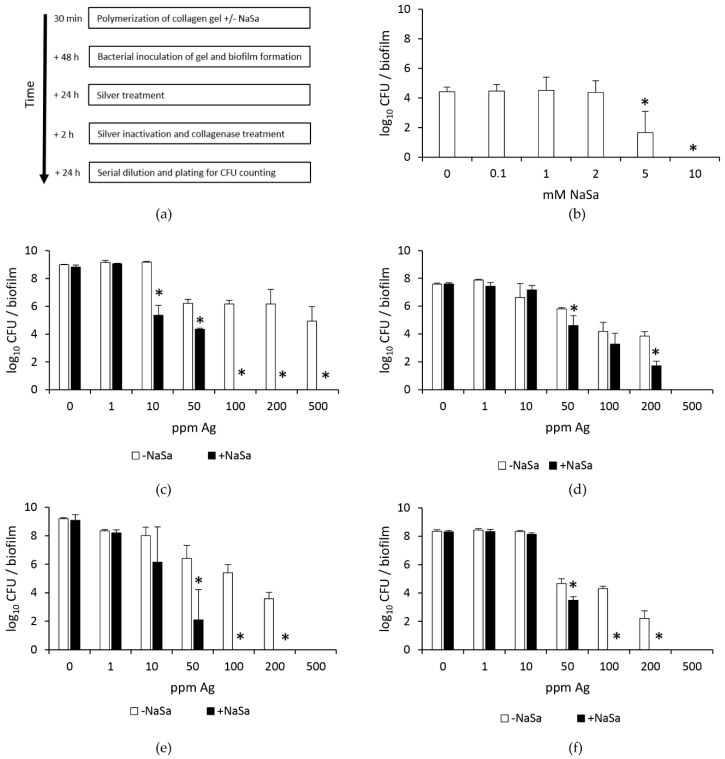
Potentiated biofilm-eradicating effect of silver (Ag) after pre-treatment with sodium salicylate (NaSa) (0–10 mM) in *P. aeruginosa* grown in a simulated wound model. (**a**) Methodological flowchart showing the steps and culture times of the 3D collagen/serum-based wound model. (**b**) Increased antibacterial effect of silver (Ag, 100 ppm) on PAO1 wt biofilms previously formed in the presence of NaSa (0–10 mM). Increased antibacterial effect of Ag (0–500 ppm) on biofilms formed in the presence of 0 and 10 mM NaSa for (**c**) PAO1 wt, (**d**) PAO1 ∆*lasR* ∆*rhlR*, (**e**) the high-virulence quorum sensing (QS) signal-producing clinical strain 5, and (**f**) the low-virulence non-QS signal-producing clinical strain 2. Mean ± SD, *n* = 3. * *p* < 0.05 using one-way ANOVA with Dunnett’s post hoc test (**b**) or Student´s two-sided *t*-test (**c**)–(**f**) compared to the control (0 mM NaSa).

The minimum inhibitory and bactericidal concentrations (MIC and MBC) of NaSa toward the commonly used laboratory strain PAO1 wt and clinical isolates (strains 2 and 5) were previously determined to be 125 and 250 mM, respectively [20]. In a 3D collagen-based biofilm model, the addition of 5 and 10 mM NaSa during the establishment of PAO1 biofilms (Figure 1a) resulted in increased biofilm susceptibility towards silver. At 100 ppm silver, the 5 mM NaSa treatment resulted in a significant 2.8 log_10_ reduction in viable cell counts, while 10 mM NaSa resulted in no viable cells (limit of detection 1.8 log_10_/biofilm) compared to control biofilms grown without NaSa (Figure 1b). Subsequently, assays were performed using NaSa concentrations fixed at 0 and 10 mM, while the amount of silver in the PAO1 test system was varied between 0 and 500 ppm. At silver concentrations of 10 and 50 ppm but not at 1 ppm, the presence of NaSa resulted in a significant increased reduction in viable cell counts (average log_10_ reductions of 3.8 and 1.9, respectively) (Figure 1c). At ≥ 100 ppm Ag, NaSa pre-treatment promoted complete bacterial killing (biofilm bactericidal concentration, BBC_NaSa+Ag_ = 100 ppm), whereas the absence of NaSa resulted in 7.9 × 10^4^–1.6 × 10^6^ CFU/biofilm (BBC_Ag_ > 500 ppm; Figure 1c). Biofilms grown in the presence of 0 and 10 mM NaSa but without silver contained 1.0 and 0.6 × 10^9^ CFU/biofilm, respectively.

Compared to treatment without NaSa, combination treatment with NaSa and silver (50 and 200 ppm) resulted in significantly reduced viability of the QS-deficient strain PAO1 ∆*lasR* ∆*rhlR* by 1.2 and 2.2 log_10_, respectively. At 500 ppm silver, no viable cells were observed in either group (BBC_NaSa+Ag_ = BBC_Ag_ = 500 ppm; Figure 1d). The NaSa-mediated increase in biofilm susceptibility to silver was also demonstrated for a highly virulent chronic wound isolate that was previously shown to produce QS signals and a range of virulence factors [20]. For this strain (strain 5), combination treatment with 10 mM NaSa and 50 ppm silver reduced cell viability by 4.3 log_10_ compared to samples treated with 50 ppm silver alone (Figure 1e). At 100–200 ppm Ag, pre-treatment with NaSa resulted in reduced cell viability below the limit of detection, whereas 2.5 × 10^5^ and 3.9 × 10^3^ CFU were detected after treatment with 100 and 200 ppm silver, respectively, in the absence of NaSa. No viable CFU were obtained from biofilms treated with 500 ppm silver (with or without NaSa). For a chronic wound isolate previously shown to produce no QS signals and very few virulence factors (strain 2) [20], NaSa significantly increased silver susceptibility at 50 ppm Ag, with an observed 1.2 log_10_ reduction in cell viability. At 100 and 200 ppm Ag, the addition of NaSa resulted in no viable CFU (below the limit of detection), whereas the absence of NaSa resulted in 2.0 × 10^4^ CFU and 1.6 × 10^2^ CFU/biofilm, respectively (Figure 1f). Taken together, these results revealed that biofilms grown in the presence of NaSa were more susceptible to silver than control biofilms, although this effect was observed to a lesser extent for the QS-mutant. For all strains tested (except PAO1 ∆*lasR* ∆*rhlR*), combination treatment with 10 mM NaSa and 100 ppm Ag completely eradicated the biofilms, reducing the MBCs of Ag by >5-fold for PAO1 wt and 5-fold for the clinical strains.

### 2.2. Silver and NaSa Suppress the Growth but Not the Viability of Pseudomonas aeruginosa

**Figure 2:**
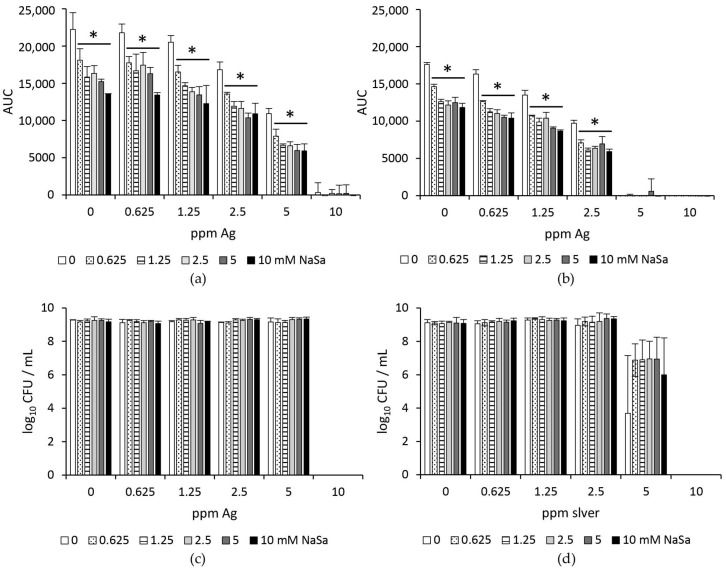
Silver (Ag) in combination with sodium salicylate (NaSa) reduces the planktonic growth but not the viability of *P. aeruginosa.* Total growth, expressed as area under the growth curve (AUC), and viability, as measured by colony-forming units (CFU) per mL, of (**a**,**c**) PAO1 wt and (**b**,**d**) PAO1 ∆*lasR* ∆*rhlR* exposed to silver (0–10 ppm) and NaSa (0–10 mM) in simulated wound fluid for 20 h. Mean ± SD, *n* = 3. * *p* < 0.05 one-way ANOVA with Dunnett´s post hoc test compared to the control (0 mM NaSa).

Growth curves from *P. aeruginosa* were generated to assess the effect of NaSa and silver on the growth potential and viability of planktonic *P. aeruginosa* cells (Figure S1). NaSa (0–10 mM) did not affect the MIC of silver (10 and 5 ppm Ag) for PAO1 wt or PAO1 ∆*lasR* ∆*rhlR*, respectively (Figure 2a,b). Although the total overall growth, as measured by the area under the growth curve (AUC), was affected by NaSa at all silver concentrations assayed below the MIC (Figure 2a,b), no difference in cell viability was observed after 20 h of cultivation for either of the strains (Figure 2c,d). Treatment with silver resulted in decreased growth potential, which was most evident at 2.5–5 ppm for PAO1 wt and 1.25–2.5 ppm for PAO1 ∆*lasR* ∆*rhlR*. However, the number of viable CFU at these silver concentrations was similar for the treated and untreated controls after 20 h of growth (Figure 2c,d).

### 2.3. NaSa Reduces Pseudomonas aeruginosa Biofilm Formation, Cell Aggregation, and Virulence Factor Production

**Figure 3:**
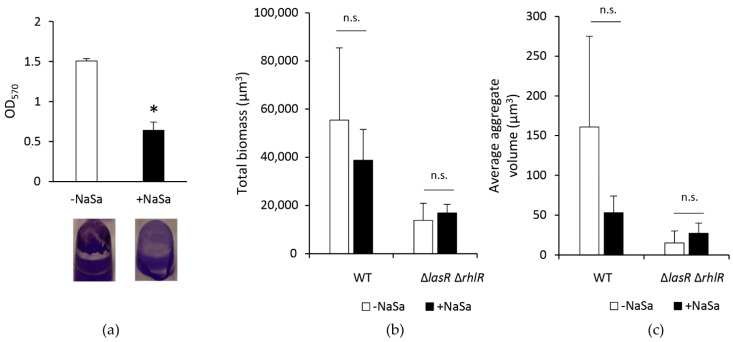
Inhibitory effect of 10 mM sodium salicylate (NaSa) on *P. aeruginosa* biofilm formation on (**a**) polystyrene pegs and (**b**,**c**) collagen-coated glass. (**a**) NaSa reduced biofilm biomass formed on polystyrene pegs by PAO1 wt grown in the presence or absence of 10 mM NaSa for 48 h using the Calgary biofilm device, as measured by mean OD of crystal violet. (**b**) Total biofilm biomass formed by PAO1 wt-GFP and PAO1 ∆*lasR* ∆*rhlR*-GFP and (**c**) average aggregate size was not affected by NaSa. Mean ± SD, *n* = 3. * *p* < 0.05 using Student´s two-sided t-test compared to the control (0 mM NaSa). n.s. is non-significant.

The increased antimicrobial effect achieved by the use of silver in combination with NaSa on biofilms led us to investigate the effect of NaSa on biofilm formation. Through crystal violet staining, PAO1 wt was observed to form 58% less biofilm biomass on polystyrene in the presence of NaSa than in its absence (Figure 3a). Collagen-coated glass was then used as a wound-like surface to analyse the 3D biofilm structures using confocal laser scanning microscopy (CLSM). The presence of NaSa in the test media did not affect the final total biofilm biomass (Figure 3b) nor the number of viable CFU (two-tailed Student’s *t*-test, *p* = 0.37 for PAO1 wt and *p* = 0.83 for PAO1 ∆*lasR* ∆*rhlR*). When comparing datasets based on the 10 largest aggregates in each image stack, the average aggregate size for PAO1 wt or PAO1 ∆*lasR* ∆*rhlR* was decreased in the presence NaSa, although not significantly (Figure 3c).

**Figure 4:**
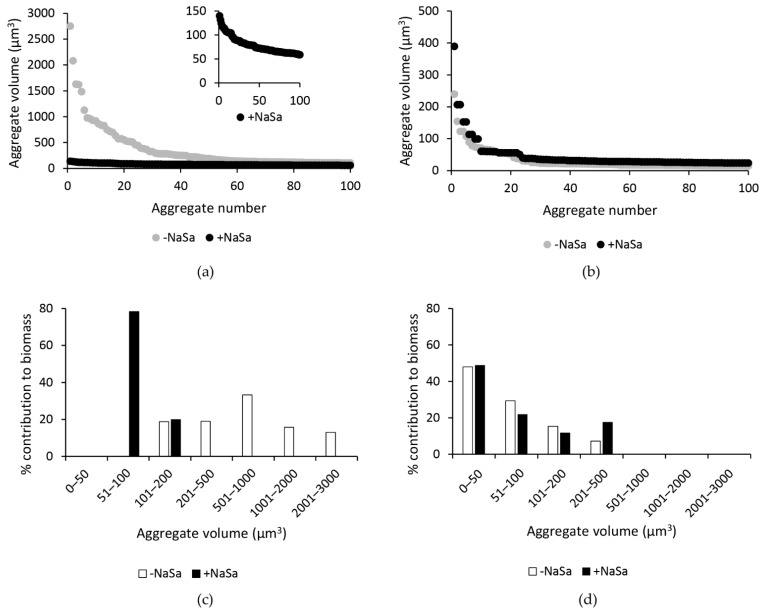
Sodium salicylate (NaSa) reduces the formation of large aggregates of PAO1 wt-GFP but not PAO1 ∆*lasR* ∆*rhlR*-GFP. Aggregate volumes of the 100 largest aggregates (selected from a dataset based on the 10 largest aggregates per z-stack), in the presence or absence of 10 mM NaSa, of (**a**) PAO1 wt-GFP and (**b**) PAO1 ∆*lasR* ∆*rhlR*-GFP. The aggregate size distribution in cultures with NaSa was narrower than that observed in cultures without NaSa of (**c**) PAO1 wt-GFP but not of (**d**) PAO1 ∆*lasR* ∆*rhlR*-GFP.

**Figure 5:**
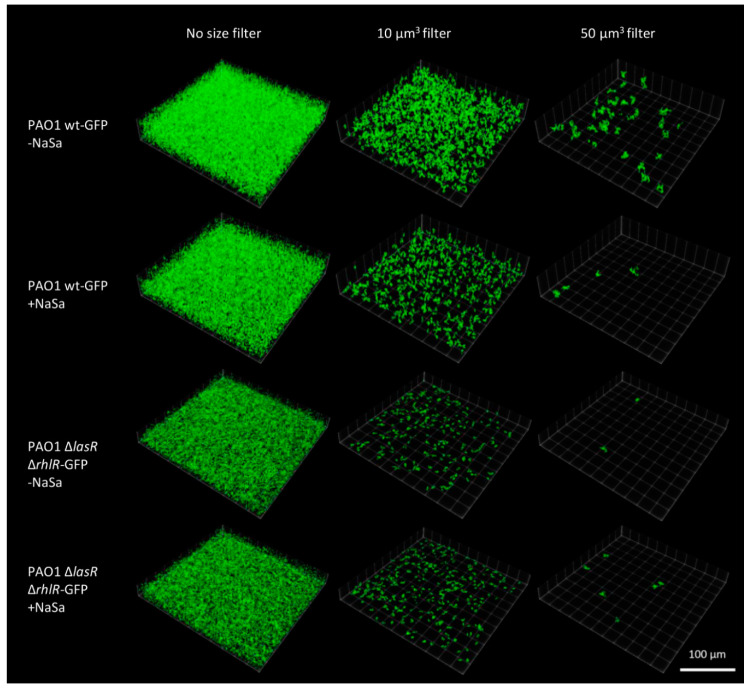
Visualization of 24 h biofilm aggregates on collagen type-I-coated glass slides. Aggregates of PAO1 wt-GFP and PAO1 ∆*lasR* ∆*rhlR*-GFP from representative image stacks, volume-filtered at 10 µm^3^ and 50 µm^3^.

However, treatment with NaSa resulted in fewer large aggregates for PAO1 wt but not PAO1 ∆*lasR* ∆*rhlR* compared to the control (Figure 4a,b). Furthermore, the size distribution of PAO1 wt aggregates was narrower in cultures with NaSa, with aggregates ranging between 0 and 100 µm^3^ accounting for 78% of the biomass in the dataset. In contrast, cultures without NaSa contained aggregates that were evenly distributed between 100 and 3000 µm^3^, with no aggregates observed between the of 0 and 100 µm^3^ (Figure 4c). For PAO1 ∆*lasR* ∆*rhlR*, aggregate sizes between 0 and 100 µm^3^ accounted for 77% of the biomass in an equivalent dataset, but in contrast to PAO1 wt, aggregate sizes were not affected by NaSa (Figure 4d). Images of representative image stacks show the differences in cell aggregation for both strains in the presence or absence of NaSa (Figure 5). Taken together, NaSa was shown to impact cell aggregation for the PAO1 wt strain but not the PAO1 ∆*lasR* ∆*rhlR* strain.

**Figure 6:**
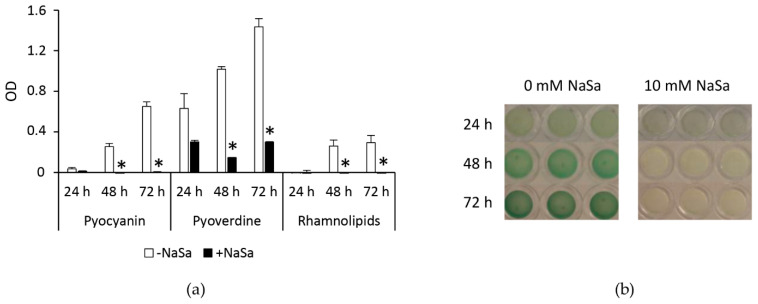
Sodium salicylate (NaSa) reduces PAO1 wt biofilm virulence factor production. (**a**) Pyocyanin, pyoverdine and rhamnolipid production in 24-, 48-, and 72-h-old biofilms formed in the presence or absence of 10 mM NaSa. (**b**) Visual appearance of the biofilms. Mean ± SD, *n* = 3 (48 h *n* = 6). * *p* < 0.05 Student’s two-sided t-test was compared to the control (0 mM NaSa) at the respective time points.

PAO1 wt was shown to produce pyocyanin, pyoverdine, and rhamnolipids during biofilm establishment (Figure 6a,b). Forty-eight hours were required to detect pyocyanin and rhamnolipids, whereas pyoverdine was detected after 24 h of cultivation. NaSa significantly reduced pyoverdine production compared to that observed in the untreated samples, by 86 and 79% after 48 and 72 h, respectively (Figure 6a). Pyocyanin and rhamnolipid production was not detected at any time point in cultures with NaSa. In simulated wound fluid (SWF) culture, presence of NaSa resulted in decreased levels of pyocyanin for both the clinical strain 5 and PAO1 compared to untreated control, while PAO1 ∆*lasR* ∆*rhlR* did not produce any detectable levels of pyocyanin (Figure S3).

## 3. Discussion

Quorum sensing (QS) influences biofilm formation and the production of virulence factors in many different bacterial species. Consequently, a number of QS-related mechanisms and potential means of interfering with these complex signalling networks have been reported [23,24]. In a previous study using wound-simulated culture conditions, we showed that NaSa attenuates virulence factor production in both laboratory strains and chronic wound isolates of *P. aeruginosa* in vitro, most likely via QS inhibition [20]. In the present study, we further investigated the influence of NaSa on *P. aeruginosa* virulence, focusing on the formation, 3D architecture and susceptibility of biofilms towards silver (a commonly used topical antiseptic). The results showed that *P. aeruginosa* biofilms formed in the presence of NaSa were significantly more susceptible to silver, as demonstrated using laboratory wild-type and chronic wound isolates.

Previous studies have revealed the increased susceptibility of biofilms grown in the presence of QS inhibitors, e.g., furanone C-30 [25] and garlic extract [26]. Importantly, the 3D collagen-based model used in the present study contained large amounts of serum proteins to better represent the protein concentration of chronic wound fluids [27,28] compared to other commonly used culture media, such as tryptic soy broth or lysogeny broth. In particular, serum albumin is known to interact with and potentially inactivate bioactive molecules [29,30], increasing the risk that they will have limited clinical efficacy. Furthermore, serum has been shown to increase QS activity and virulence factor production [20,31], while albumin alone reduces QS, potentially by sequestering QS signals [32].

In the absence of silver, NaSa alone did not affect the viability of the biofilms for any of the tested strains. Using the 3D serum-collagen model, 5 mM NaSa was shown to be required to promote a significant increase in PAO1 susceptibility towards silver. For the combined 10 mM NaSa and 100 ppm silver treatment, a 100% bactericidal effect was observed for PAO1 wt and the clinical isolates. Interestingly, the presence of NaSa during biofilm formation, followed by silver treatment, also resulted in reduced colony counts for the QS-deficient PAO1 strain and the low-virulence non-QS signal-producing clinical strain 2. However, considering that biofilms from the wild-type strain were more resistant to silver than those from the QS mutant, NaSa pre-treatment of biofilms decreased the BBC for PAO1 wt by more than 5-fold compared to the PAO1 QS mutant with an unchanged BBC. These results, which show that the mutant was less affected by NaSa than the wild-type strain, support earlier findings that NaSa inhibits QS [20]. To a lesser extent, NaSa had an effect on the susceptibility of the QS mutant to silver, indicating that factors other than QS inhibition alone may also play a role in this process, e.g., decreasing total growth. This assumption is in line with observations that salicylates affect bacteria in different ways [33], including promoting decreased metabolism in *E. coli* [34] and causing changes in the membrane proteins of *P. aeruginosa*, including the two fimbrial proteins PilQ and flagellin type-A, which can affect bacterial adhesion [35].

Since no differences were observed in the total viable CFU from biofilms or planktonic cultures grown with or without NaSa, NaSa was hypothesized to instead affect other biofilm properties. In support of this idea, the total biofilm biomass formed on polystyrene pegs was significantly lower when biofilms were grown in the presence of NaSa, as measured by the absorbance of crystal violet, which binds both to cells and extracellular polymeric substances (EPS) [36]. The results of the present study contrast with those of our recent study, which showed that NaSa did not affect PAO1 wt biofilm formation on polystyrene [20]. Methodological differences are likely to explain this difference, as in the previous study the inoculum cells from the overnight culture were not washed prior to the addition of NaSa, potentially resulting in an abundance of QS signal molecules and virulence factors being present at the start of the experiment.

To understand whether the NaSa-mediated increased biofilm susceptibility towards silver could be due to changes in biofilm architecture, confocal laser-scanning microscopy (CLSM) was used. A collagen type I-coated glass surface was used to image the biofilms to overcome the instability of the 3D collagen biofilm model due to gel degradation after 48 h of cultivation (possibly via the production of collagen-degrading enzymes). The image analysis results indicated that aggregate volumes were smaller and more narrowly distributed in PAO1 wt cultures with NaSa than that observed in those without NaSa. In contrast, NaSa did not affect cell aggregation in biofilms of the PAO1 ∆*lasR* ∆*rhlR* mutant, which contained smaller and more evenly size-distributed clusters compared to the wild-type strain, indicating that the NaSa-dependent inhibition of cell aggregation is QS-mediated.

Increased antimicrobial tolerance has previously been shown to be dependent on biofilm thickness [37], indicating that the observed NaSa-mediated increase in silver susceptibility could be aggregate size-dependent. Several characteristics, such as poor antimicrobial penetration into the biofilm, binding and inactivation of the antimicrobial agent by EPS components, and the presence of highly tolerant persister cells, are all involved in the antimicrobial resistance of biofilms [38,39]. Using NaSa to interfere with QS and virulence could affect the components and architecture of biofilms in different ways. The expression of pyocyanin, a key virulence factor of *P. aeruginosa*, is tightly controlled by different QS subsystems [40]. A *P. aeruginosa* mutant defective in pyocyanin production forms biofilms with less biomass and thickness than those formed by the corresponding wild-type strain, a phenotype that can be reversed by adding exogenous pyocyanin [41]. Extending previous observations that NaSa reduces pyocyanin production in serum-containing cultures of *P. aeruginosa* [20], a similar effect was demonstrated in the biofilm model used in the present study. In contrast to biofilms formed in the absence of NaSa, those treated with NaSa produced undetectable levels of pyocyanin, which could have contributed to the smaller aggregate sizes. Other potential mechanisms of NaSa activity include alterations in the QS-regulated virulence factors rhamnolipids and pyoverdine, which are both involved in regulating biofilm architecture and channel formation within biofilms [42,43,44]. In the present study, the presence of NaSa during biofilm establishment resulted in undetectable levels of rhamnolipids over a 3-day culture period. Similarly, pyoverdine levels were significantly reduced at all tested time points in NaSa-containing cultures, which could have played a role in the observed changes in biofilm structure and silver susceptibility. Based on these observations, and in agreement with previous work [20], the reduced biofilm aggregation of PAO1 wt caused by NaSa treatment could have been due to the inhibition of QS-related gene expression and reduced pyocyanin, rhamnolipid, and pyoverdine production. However, the regulation of biofilm formation in *P. aeruginosa* is highly complex, and multiple mechanisms apart from QS also play a role [45].

The total growth, measured as the area under the growth curve (AUC), was significantly decreased by NaSa treatment, with a flatter growth curve (slower growth rate) observed compared to the control biofilm. However, no significant NaSa-dependent difference, with or without Ag treatment, was observed in the number of viable CFU after 20 h of treatment. To evaluate a potential partial reduction in PAO1 wt viability in the presence of NaSa, other silver concentrations (6 and 7.5 ppm) were tested. A silver concentration of 7.5 ppm resulted in complete killing at all NaSa concentrations, whereas, at 6 ppm silver and across all NaSa concentrations, viability was not affected (Figure S2). The finding that NaSa does not potentiate the effect of silver against planktonic cells supports the hypothesis that the primary effect of NaSa is attributed to QS inhibition. Aspirin, which is closely related to NaSa, has previously been shown to both decrease and increase the MICs of various antibiotics, highlighting the importance of selecting the most suitable antimicrobial agent for use in combination treatment with NaSa [46].

A limitation of this study is the lack of multiple wound isolates. Although we show that NaSa reduces virulence factor production and aggregation in PAO1 biofilms, this might not be directly translatable to clinical isolates. It has been shown that in chronic infections in cystic fibrosis (CF) patients, mutations of the lasR receptor may occur, whereas rhlR is less affected [47]. Similarly, chronic wound isolates defective in elastase production have been identified [20,48] although data indicate that phenotypic heterogeneity might be less extensive in chronic wounds than in CF infections [49]. Hence, QS-inhibition of the rhlR system might be more effective clinically. We have previously shown that NaSa reduces QS activity of both lasR and rhlR using *lasB* and *rhlA* reporter systems [20] but the molecular target interaction of NaSa has not been further investigated.

For all new treatment strategies, potential cytotoxic or other adverse effects, such as repression of the immune system, need to be considered and weighed against the therapeutic benefits. Silver possesses cytotoxic effects but is considered safe at occupational exposure and regarded useful in the treatment of infected wounds [50,51]. From a biocompatibility perspective, the findings in the present study that the amount of silver needed to eradicate a biofilm was significantly lower in presence of NaSa, indicate that a combination treatment would be beneficial. Although toxicity and tolerance of salicylates have been intensively studied [52,53] due to the routinely use of acetylsalicylic acid for anti-inflammatory purposes, the safety of the use of salicylates on wounds needs to be investigated.

In conclusion, the results of the present study showed that biofilms, but not planktonic cells, were more susceptible to silver when grown in the presence of NaSa, reducing the minimum biofilm bactericidal concentration more than 5-fold in QS-competent *P. aeruginosa* strains. NaSa appeared to decrease biofilm aggregation and aggregate size distribution in QS-competent but not in QS-deficient *P. aeruginosa* strains. A plausible mode of action of NaSa include QS inhibition with a reduction of EPS components, resulting in decreased biofilm formation, with no bactericidal effect on planktonic and biofilm cells. These findings on combining NaSa and silver treatment should encourage further evaluation to assess its potential as a treatment for wounds infected with *P. aeruginosa*.

## 4. Materials and Methods

### 4.1. Pseudomonas aeruginosa Strains and Culture Conditions

The *P. aeruginosa* strains used in the study are summarized in Table 1. Strains stored at −80 °C in cryotubes containing Tryptone Soya Broth (TSB) and glycerol were subsequently cultured overnight at 37 °C on 5% horse blood Columbia agar plates (Medium Department, Clinical Microbiology Lab, Sahlgrenska University Hospital, Gothenburg, Sweden) before inoculum preparation in different media. All chemicals were obtained from Sigma-Aldrich (St. Louis, MO, USA) unless otherwise stated.

### 4.2. Pseudomonas aeruginosa Biofilm Formation and Susceptibility Towards Silver in the Presence or Absence of NaSa

To evaluate biofilm formation on polystyrene, the Calgary biofilm device (Innovotech, Edmonton, Canada) and crystal violet staining [57] were performed as described previously [20], with the modification that cells harvested from overnight culture plates were washed twice in 0.9% saline (NaCl) solution and centrifuged for 10 min at 4000 *g* prior to resuspension. The data are presented as the average of three independent experiments, each of which was performed with three replicates. To mimic wound-like conditions, an in vitro biofilm wound model [22] was established by mixing ice-cold rat tail collagen type 1 (10 mg/mL; Corning, NY, USA) with 0.1% HAc, 50% non-heat inactivated foetal bovine serum (HyClone Laboratories, Logan, UT, USA) in saline and 0.1 M NaOH at a 2:1:6:1 ratio. Fifty microlitres of the prepared collagen-serum solution was added to each well of sterile flat-bottomed 96-well polystyrene plates (Nunc, Roskilde, Denmark) and allowed to solidify to a gel at 35 °C in a humidified incubator for 30 min. For samples containing sodium salicylate, NaSa was added to the prepared collagen-serum solution prior to its addition to the wells. Overnight cultures of PAO1 wt, PAO1 ∆*lasR* ∆*rhlR*, and two *P. aeruginosa* chronic wound strains (strain 2 = M20.447 and strain 5 = M20.450) on blood agar were washed twice in saline by centrifugation at 4000 *g* for 10 min and then resuspended to OD_546_ = 0.1 in saline supplemented with 50% (*v/v*) foetal bovine serum (simulated wound fluid, SWF) before being further diluted 1:10 in the same medium. Five microlitres of the inoculum was added to the gels, which were then incubated at 35 °C for 48 h to promote biofilm formation. Then, 20 µL of silver sulphate dissolved in SWF was added on top of the biofilm-containing gels, and the gels then were further incubated at 35 °C for 24 h. Final silver concentrations in the wound model ranged between 0 and 500 ppm. After silver treatment, the antimicrobial effect was terminated by degrading the gels in 200 µL of Dey-Engley (DE) neutralization solution and 33 µL of collagenase (3 mg/mL) from *Clostridium histolyticum* in a buffer comprising 130 mM NaCl, 10 mM CaAc, and 20 mM HEPES. After incubating for 2 h at 35 °C, the dissolved gels were collected and serially diluted 10-fold in DE followed by spot plating on blood agar plates and incubation at 35 °C for 24 h. The number of colonies was manually counted. The data are presented as the average of three independent experiments, each of which was performed with two technical replicates.

### 4.3. Pseudomonas aeruginosa Biofilm Virulence Factor Production

To assess pyocyanin, pyoverdine and rhamnolipid production, PAO1 wt biofilms were formed using the 3D collagen-gel model as described previously in the presence or absence of 10 mM NaSa but without silver for 24, 48, or 72 h prior to enzymatic degradation of the gel using 100 µL of collagenase (0.15 mg/mL in saline). The supernatants of the degraded gels were collected by centrifugation at 14,000 *g* for five minutes. For pyocyanin and pyoverdine measurements, 50 µL of supernatant was transferred to a 384-well plate, and the blank-corrected absorbance was read at 690 and 405 nm, respectively. For rhamnolipid production [58], supernatants of the technical replicates were pooled prior to mixing 300 µL of supernatant with 1.7 mL of diethylether (VWR, Radnor, PA, USA). The mixture was then vortexed and centrifuged at 2000 *g* for one minute, after which the ether fractions were collected in Eppendorf tubes and evaporated overnight at room temperature. Subsequently, 200 µL of dH_2_O was added to the tubes and the samples were vortexed, after which 100 µL of each suspension was added to 900 µL of 0.19% (*w/w*) orcinol in 53% (*w/w*) H_2_SO_4_. The mixture was then incubated at 80 °C for 30 min before 50 µL was transferred to a 384-well plate, and the absorbance was measured at 421 nm. The data are presented as the average of three separate experiments, each of which was performed with four technical replicates. For rhamnolipid production, six separate experiments were performed for the 48-h time point. Uninoculated gels were used as blank samples. Pyocyanin production in serum-containing broth (LB supplemented with 50% foetal bovine serum) culture was performed using clinical strain 5, PAO1, and PAO1 ∆*lasR* ∆*rhlR* as described previously [20] with the modifications of using a culture time of 72 h and NaSa concentrations of 0 and 10 mM.

### 4.4. Planktonic Cells of Pseudomonas aeruginosa

A checkerboard approach was used to investigate the combinatory effect of NaSa and silver on planktonic PAO1 wt and ∆*lasR* ∆*rhlR* cells with respect growth and antimicrobial effects. In a 96-well plate, 20 mM NaSa was added to the first column of six rows and serially diluted column-wise (1:2, five times) in SWF, where the sixth column contained only SWF. Similarly, 20 ppm Ag in SWF was added to the first row of six columns and serially diluted row-wise (1:2, five times), where the sixth row contained only SWF. Next, the two dilution series were mixed 1:1 with each other, creating a pattern with 36 unique Ag-NaSa combinations. All SWF used contained a 1:100 dilution of an OD_546_ = 0.1 inoculum in saline, resulting in a final bacterial density of 10^6^ CFU/mL. Subsequently, 50 µL of each combination was added to 384-well plates (Nunc). The plates were placed in a plate reader at 35 °C, and kinetic absorbance readings at 600 nm were recorded every 30 min for 20 h. Total overall growth for the different treatment groups was determined by calculating the area under the growth curve (AUC) of blank-corrected data. After the final OD reading, the number of viable counts per well was determined by standard CFU counting on blood agar plates. Three independent experiments were carried out with single samples.

### 4.5. Pseudomonas aeruginosa Biofilm Architecture

Overnight colonies of PAO1 wt-GFP and PAO1 ∆*lasR* ∆*rhlR*-GFP on blood agar were washed twice in saline by centrifugation at 4000 *g* for 10 min, resuspended to OD_546_ = 0.1 in SWF and then diluted 1:100 in the same medium with and without 10 mM NaSa. Subsequently, 500 µL of inoculum was added to the wells of rat tail collagen type-I-coated glass chamber slides (Corning) and statically incubated at 35 °C for 24 h. The medium in the wells was discarded before removing the chamber wall construct. Then, a glass coverslip was gently placed on top of the surface, and image z-stacks (211 × 211 × 40 µm, L × W × H, 1 µm step size at a resolution of 512 × 512 pixels) were collected using a Nikon C2 confocal laser-scanning microscope (Nikon, Tokyo, Japan) with a 488 nm laser and a 60× water immersion objective (NA 1.20). Using position unbiased selection approach, five image stacks were collected from each duplicate sample (from three independent experiments), yielding 20 image z-stacks per treatment. Data files (.ND2) were imported into ImageJ 1.53c [59] (Fiji package), and z-stacks were segmented and made binary using Otsu’s thresholding method. Aggregates were identified using the 3D object counter plugin [60] with a minimum cell size of 0.46 µm^3^ (volume of rod-shaped *P. aeruginosa* cells with a diameter of 0.5 µm and length of 2.3 µm). The 10 largest aggregates in each z-stack were pooled into a new data set, resulting in 300 aggregates per strain and treatment, which were further analysed and compared regarding size. To visualize differences in aggregate size, aggregates of representative samples for PAO1-GFP wt and PAO1 ∆*lasR* ∆*rhlR*-GFP were filtered using 10 and 50 µm^3^ filters, and the ClearVolume plugin for ImageJ was used to visualize the aggregates in 3D [61].

## Figures and Tables

**Table 1:** Pseudomonas aeruginosa strains used in this study.

Strain Name	Origin and Properties	Reference
2 (M20.447) 5 (M20.450)	Clinical wound isolates of *P. aeruginosa,* with strain 2 and 5 exhibiting low and high in virulence factor and QS signal production, respectively	[20,54]
PAO1 wt	Reference *P. aeruginosa* strain (Pseudomonas Genetic Stock Center; strain PAO0001)	
PAO1 ∆*lasR* ∆*rhlR*	PAO1 lacking the QS signal receptors *lasR* and *rhlR*	[55]
PAO1 wt-GFP	PAO1 tagged with eGFP in a mini-Tn7 construct, Gmr	[56]
PAO1 ∆*lasR*∆*rhlR*-GFP	PAO1 ∆*lasR* ∆*rhlR* tagged with GFP expressed on plasmid pMRP9	[55]

## Data Availability

All datasets supporting the conclusions of this article are included in the article and supplementary file.

## Supplementary Materials

Supplementary materials is available at https://www.mdpi.com/1422-0067/22/3/1060/s1.

## Abbreviations

Ag	Silver
ASA	Acetylsalicylic acid
BBC	Biofilm bactericidal concentration
CF	Cystic fibrosis
CFU	Colony forming units
CLSM	Confocal laser scanning microscopy
eDNA	Extracellular DNA
EPS	Extracellular polymeric substances
MBC	Minimum bactericidal concentration
MIC	Minimum inhibitory concentration
OD	Optical density
SA	Salicylic acid
SWF	Simulated Wound Fluid
NaSa	Sodium salicylate
QS	Quorum sensing
QSIs	Quorum sensing inhibitors
