# Supplementary material for: Sodium Salicylate Influences the Pseudomonas aeruginosa Biofilm Structure and Susceptibility Towards Silver
Source: Int J Mol Sci. 2021 Jan 21;22(3):1060. doi: 10.3390/ijms22031060 (PMC7865925; doi:10.3390/ijms22031060)
Supplement: Supplementary file 1 [file ijms-22-01060-s001.pdf]

International Journal of *Molecular Sciences*

MDPI

Supplemental Material

Sodium Salicylate Influences the *Pseudomonas aeruginosa* Biofilm Structure and Susceptibility Towards Silver

Erik Gerner[1,2,3,*], Sofia Almqvist[2], Peter Thomsen[1], Maria Werthén[1,3], and Margarita Trobos[1,3,*]

[1] Department of Biomaterials, The Sahlgrenska Academy, University of Gothenburg, Gothenburg, Sweden; erik.gerner@biomaterials.gu.se (E.G.); peter.thomsen@biomaterials.gu.se (P.T.); werthenm@gmail.com (M.W.); margarita.trobos@biomaterials.gu.se (M.T.)

[2] Mölnlycke Health Care AB, Gothenburg, Sweden; sofia.almqvist@molnlycke.com (S.A.); erik.gerner@molnlycke.com (E.G.)

[3] Center for Antibiotic Resistance Research (CARe) at University of Gothenburg, Gothenburg, Sweden

* Correspondence: margarita.trobos@biomaterials.gu.se (M.T.); erik.gerner@biomaterials.gu.se (E.G.)

Received: 10 December 2020; Accepted: 19 January 2021; Published: 21 January 2021

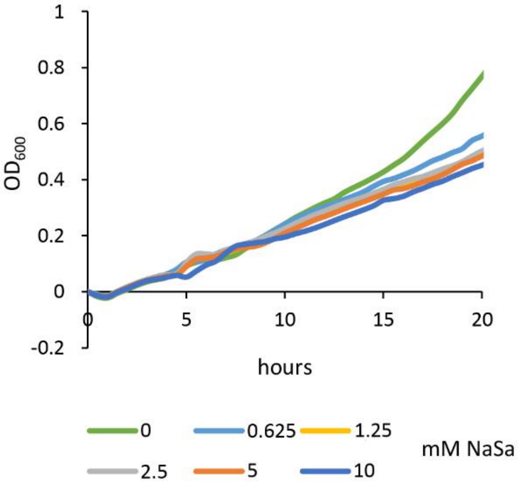
OD600
hours
0
0.625
1.25
2.5
5
10
mM NaSa

(a)

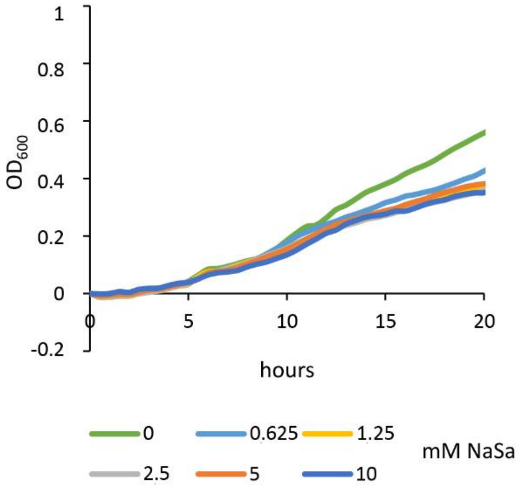
OD600
hours
0
0.625
1.25
2.5
5
10
mM NaSa

(b)

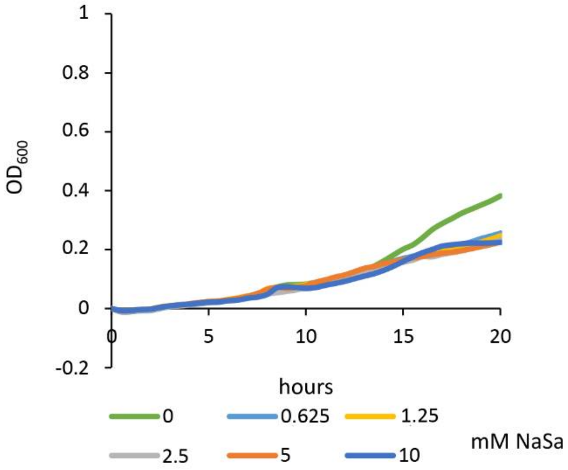
OD600
hours
0
0.625
1.25
2.5
5
10
mM NaSa

(c)

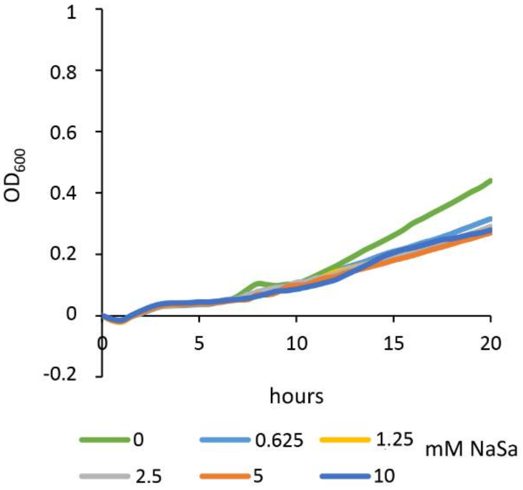
OD600
hours
0
0.625
1.25
2.5
5
10
mM NaSa

(d)

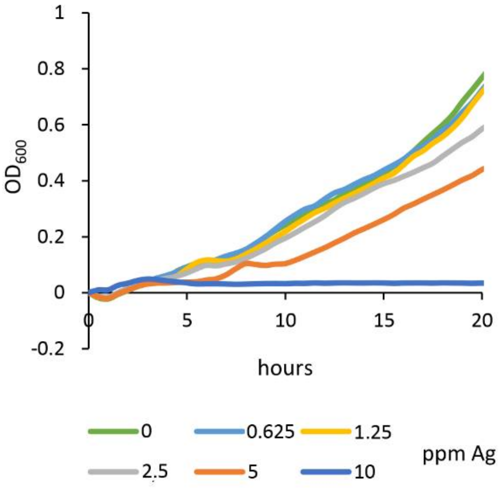
OD600
hours
0
0.625
1.25
2.5
5
10
ppm Ag

(e)

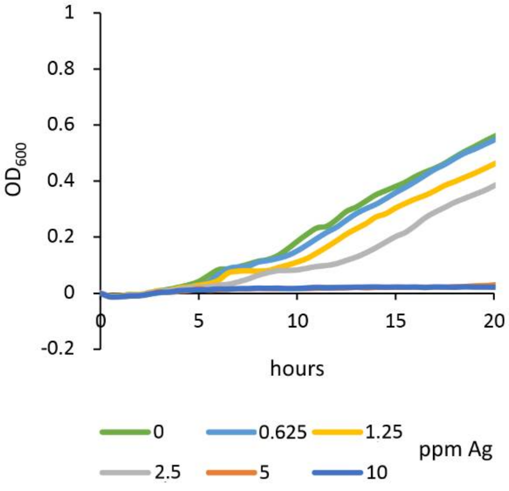
OD600
hours
0
0.625
1.25
2.5
5
10
ppm Ag

(f)

Figure S1. Growth curves of (**a, c** and **e**) PAO1 wt; and (**b, d** and **f**) PAO1 *lasR* Δ*rhlR* cultured in simulated wound fluid in the presence of (**a-b**) 0-10 mM NaSa without Ag; 0-10 mM NaSa with (**c**) 5 or (**d**) 2.5 ppm Ag and (**e-f**) in 0-10 ppm Ag without the addition of NaSa. N = 3.

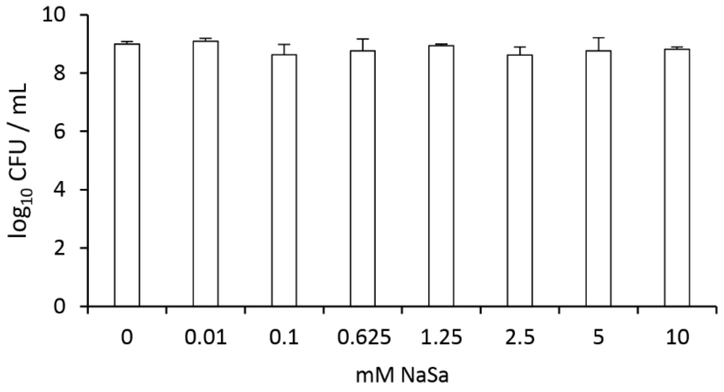

Figure S2. Silver (Ag) in combination with NaSa does not reduce the viability of planktonic cultures of *P. aeruginosa*. Viability, measured as colony-forming units (CFUs), of PAO1-wt exposed to 6 ppm silver and NaSa (0-10 mM) in simulated wound fluid for 20 h. At 7 ppm Ag, total killing in both the control and across all NaSa concentrations was observed. Mean ± SD, n = 3.

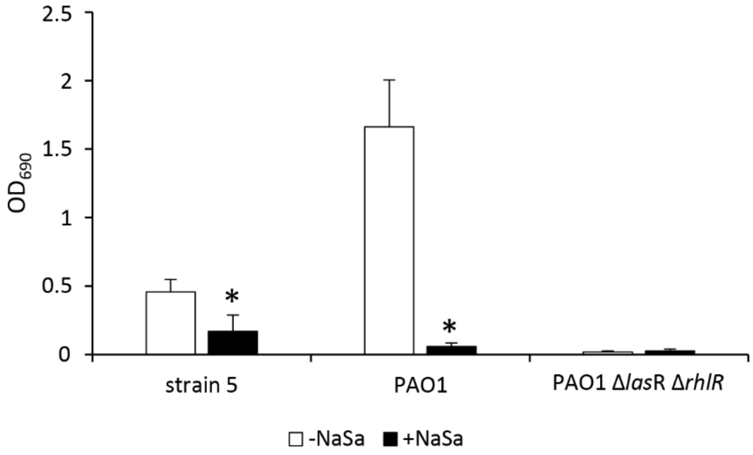

Figure S3. NaSa reduces pyocyanin production in serum-containing broth culture of strain 5 and PAO1, but not of PAO1 PAO1 lasR ΔrhlR. *$p<0.05$ Student's two-sided T-test compared to control (0 mM NaSa).
